# Adsorption of Pb^2+^ by ameliorated alum plasma in water and soil

**DOI:** 10.1371/journal.pone.0210614

**Published:** 2019-01-25

**Authors:** Ningyu Li, Qinglin Fu, Bin Guo, Chen Liu, Hua Li, Yongzhen Ding

**Affiliations:** 1 Institute of Environment, Resource, Soil and Fertilizer, Zhejiang Academy of Agricultural Sciences, Hangzhou, China; 2 Agro-Environmental Protection Institute, Ministry of Agriculture, Tianjin, China; University of Eastern Finland, FINLAND

## Abstract

Four methods, including hot acid treatment, hot alkali treatment, calcination treatment and sulfhydrylation treatment, were applied to activate alum plasma in order to obtain new Pb^2+^ adsorbents. The corresponding adsorption isotherm satisfies the Langmuir equation, and the maximum adsorption of the alum plasma after hot acid treatment, hot alkali treatment and high-temperature calcination were 18.9, 57.3 and 10.9 mg·g^−1^, respectively, and in the range of 1.23–6.57 times greater than the adsorption capacity of the original alum plasma. The soil culture experiments indicated that the effective Pb content in the soils treated with hot alkali ameliorated alum plasma was significantly lower (p < 0.05) than those treated with the other three types of alum plasma. For example, if the additive content is 5.0%, after a storage period of 16 weeks, the effective Pb content becomes 19.87 mg·kg^−1^, which corresponds to a reduction of 60.9% in comparison with the control sample. In addition, Specific surface area (BET), Scanning electron microscopy (SEM) and Fourier transform infrared spectroscopy (FIR) were used to characterize the microstructure of alum plasma before and after amelioration. It was evident that hot alkali treatment of alum plasma resulted in smaller particles, a significantly higher specific area and lower mineral crystallinity, which improved the adsorption performance of Pb^2+^. In conclusion, hot alkali treatment of alum plasma indicates relatively good Pb^2+^ adsorption ability, and is a promising novel adsorbents that could ameliorate soils that have been polluted by heavy metal Pb.

## Introduction

Lead (Pb) is one hazardous pollutant that displays strong toxicity in soils, and is an important restricted element according to the safety standards of agricultural products. It exists in soil in various forms, including a water-soluble state, ion-exchangeable state, organically bound state, carbonate bounded state and a residue state. The adsorption of Pb by plants in the soil primarily depends on the concentrations of water-soluble Pb and ion-exchangeable Pb [1]. The addition of a passivant could absorb/immobilize water-soluble Pb in the soil, thus lowering its accumulation in plants and animals, for example, in phosphate materials, alkaline matters, biochar and red mud, amongst other [2–6]. Following the introduction of adsorbents, the exchangeable, high-activity Pb in the soil transforms into a residual state that is low in activity. As a result, the biological effectiveness and mobility of Pb is effectively lowered [7]. However, the different types of adsorbents have their own application scopes and limitations. For instance, calcareous materials might cause soil organics to decompose rapidly, hampering the accumulation of humus and leading to the deterioration of soil structures. Phosphorus-bearing matters are typically used in acidic soils, and an overdose could cause water eutrophication, resulting in secondary pollution [8,9]. As a consequence, the selection and synthesis of adsorbents that consume few resources and energy, along with limited environmental disturbance, has become a prominent research topic.

Alum plasma is composed of crystalline and amorphous silicas or silicon aluminate clay minerals formed by silicone oxide, aluminum oxide and water, in which there are abundant macro-elements including Ca, Mg, Fe, K, S and P, as well as a variety of micro-elements [10]. We have found that the basic structural unit of alum plasma is a silicon-oxygen tetrahedron and an aluminum-oxygen octahedron, which possess relatively stable chemical properties. Upon grinding, characteristics such as fine particles, good dispersion and large specific area are observed. The unique surface chemical properties of alum plasma confer its strong adsorption capability of heavy metal Pb, which influences the ability of soils to adsorb (desorb) Pb and dissolve (deposit) Pb. It alters the Pb concentration in a soil solution, determines the solid-liquid ratio distribution, and further influences the concentration, form, chemical behavior and bioavailability of Pb in soils. Evidently, alum plasma indicates an ability to absorb heavy metal ions.

Though alum plasma reveals certain affinity for absorbing and immobilizing heavy metal ions, it is still not efficient enough to directly restore polluted soils. For instance, there are many hollow structures inside the alum plasma particles as well as many impurities clogging the channels. To improve the performance of alum plasma in absorbing and immobilizing heavy metal ions, its micropore configuration needs to be strengthened, and the surface properties of the minerals need to be activated. Consequently, this paper investigated four amelioration methods to activate the treatment of raw alum plasma in order to obtain new adsorbents. These included: hot acidic treatment, hot alkali treatment, calcination treatment and sulfhydrylation treatment. The effective functional groups in alum plasma, and the effects of inorganic mineral components on Pb^2+^ absorbance, were explored by comparing the properties (dynamic, adsorption isotherm) of meliorated alum plasma in adsorbing Pb^2+^ and studying the influencing factor pH, taking IR spectrum into consideration. Furthermore, soil culture experiments were conducted, and the content difference of exchangeable Pb lowered by ameliorated alum plasma in soils was compared such that the best ameliorated alum plasma could be identified, providing experimental support for the application of ameliorated alum plasma in restoring soils polluted by Pb.

## Materials and methods

### Test materials

The alum plasma sample was obtained from an alum mine located in Wenzhou, China. The sample was washed for neutralization, grinded and passed through a 100-mesh sieve. After being dried at 65°C, the sample was considered to be original alum plasma (T0). The original alum plasma was then treated using the following four treatment methods:

Hot acidic ameliorated alum plasma (T1): the sieved alum plasma was mixed into a l mol·L^−1^ HCl solution in order to prepare an alum pulp solution using a l:10 solid-liquid ratio (g·mL^−1^). The mixed solution was then heated in a water bath at 80°C for 2 h. After completing the heat treatment, distilled water was applied several times to remove chloride ions until no white precipitate was observed upon application of AgNO_3_ detection solution. The solution was maintained at room temperature, and the supernatant was removed after the alum pulp precipitated. The beaker was then placed in an oven at 65°C to dry [11,12].

Hot alkali ameliorated alum plasma (T2): the sieved alum plasma was mixed into a l mol·L^−1^ NaOH solution in order to prepare an alum plasma solution using a l:10 solid-liquid ratio (g·mL^−1^). The solution was heated in a water bath at 80°C for 2 h. Following heat treatment, distilled water was applied to neutralize the pH of the alum plasma. The beaker containing the solution was maintained at room temperature, and the supernatant of the solution retrieved after the alum pulp precipitates were removed. The beaker was then placed in a dryer at 65°C for future use [13,14].

High-temperature calcinated alum plasma (T3) was produced in a muffle furnace, and the alum plasma was calcinated at 800°C for 2 h. Following calcination, the alum plasma was cooled to room temperature, and after being neutralized with water, it was dried and grinded, and passed through a 100-mesh sieve for future use [15,16].

Sulf-hydrylated alum plasma (T4) was prepared by adding 200 mL thioglycolic acid and 140 mL acetic anhydride into a conical flask with a cover. Five droplets of sulfide acid were later added, the solution was mixed, and 200 g of alum plasma was passed through a 100-mesh sieve. The solution was mixed, covered and placed in a dryer at 80°C for 24 h. It was then filtered, neutralized with water to remove the remaining thioglycolic acid, and naturally dried without direct sunlight. Finally, the solution was stored in a well-sealed, cool, dark container for future use [17].

### Batch adsorption test

The adsorption experiment was performed at room temperature (25 ± 0.5°C), with a horizontal interlaced image oscillation of 110 r·min^−1^. The lead nitrate solution was prepared using a 0.01 mol·L^−1^ NaNO_3_ background solution. The adsorbent value was 5 mg of sample/20 mL lead nitrate solution. The pH of the solution was adjusted using 0.1 mol·L^−1^ HCl and 0.1 mol·L^−1^ NaOH solutions. The initial concentration of lead nitrate used in the dynamic experiments was 20 mg·L^−1^ (calculated using the concentration of Pb^2+^; the adsorbent amount was also calculated using Pb^2+^), the initial pH of the solution was 5.5 and nine measurement periods were selected (1.0–36 h). The initial lead nitrate concentration used in the adsorption isotherm was within the range of 0–100 mg·L^−1^, the pH of the solution was 5.5, six concentration points were selected and the equilibrium time was 24 h. In the pH influenced experiments, 10 points encompassing a pH between 1.0–10.0 were selected and the initial lead nitrate concentration was 20.0 mg·L^−1^. Following equilibrium, the sample was high-speed centrifuged for 10 min. The supernatant was filtered using a0.45 μm Millipore filter. An inductively coupled plasma source mass spectrometer (ICP-MS, Agilent Technologies Co. Ltd. USA) was used to measure the Pb^2+^ concentration in the filtrate. The absorbent amount and absorbent rate were calculated using the concentration difference between the initial concentration and the concentration at equilibrium. A blank sample was also measured. According to the concentration difference of Pb^2+^ in the solution before and after equilibrium, the absorbent amount was calculated. The absorbent amount *Q* at equilibrium is calculated using the following equation [18,19]:

$$Q=V\left(C_{0}–C_{e}\right)/m$$ (1)

where *Q* is the Pb^2+^ weight (mg·g^−1^) absorbed in unit weight after absorption equilibrium, *C*_0_ is the initial Pb^2+^ concentration in the solution (mg·L^−1^), *C*_e_ is the final Pb^2+^ concentration in the solution (mg·L^−1^), and *V* is the volume of the solution that contains Pb^2+^ (mL), *m* is the absorbent weight (g).

### Soil culture experiment

Experimental soil in this study included farmland and vegetable garden soil obtained from Yinshan Mine, Shangyu city of Zhejiang Province. The superficial soil layer (0–20 cm) was collected, dried indoors, grinded, mixed and passed through a 40-mesh sieve. The pH of the soil was determined to be 6.71, the organic matter content was 19.5 g·kg^−1^ and the total contents of nitrogen, phosphorus and potassium were 1.26 g·kg^−1^, 0.9 g·kg^−1^ and 30.7 g·kg^−1^, respectively. The Pb content was 721.3 mg·kg^−1^.

Next, 50 g of in situ contaminated soil was placed in a 100 mL plastic beaker, and the five types of alum plasma (T0: original alum plasma, T1: hot acidic alum plasma, T2: hot alkali alum plasma, T3: high-temperature calculated alum plasma and T4: sulf-hydrylated alum plasma) at 0, 0.1%, 0.2%, 0.5%, 1%, 2% and 5% concentrations were added and fully mixed with the soil. Experiments were carried out in a culture room at a constant temperature of 25 ± 1°C. Every other day, the soil was supplied with a certain amount of deionized water such that the soil moisture reached around 60% of the field capacity. The passivation period was four, eight and 16 weeks. For each treatment, three replicates were conducted, and the effective content of heavy metal Pb in soils was measured.

The extraction method of effective Pb was as follows: firstly, the dried soils were sieved using a 2 mm mesh. Then, 5 g of sieved soils were then placed in a 50 mL centrifugal tube and 25 mL extracting agent (0.1 mol·L^−1^ CaCl_2_) was added at room temperature (−25°C), after which the beaker was placed on a horizontal oscillator and oscillated for 2 h. Following centrifugation, the supernatant was analyzed using an ICP-MS (Agilent Technologies Co. Ltd., USA).

### Measurements and methods

Structural characterizations, including particle size and specific surface area, were carried out for the ameliorated alum plasma. Microscopic images of the samples were obtained using a HITACHI S23000N Scanning Electron Microscopy (SEM) with a voltage of 30 kV. The specific surface area was measured according to the BET nitrogen adsorption method using an Accelerated Surface Area and Porosimetry system (ASAP2000). The infrared spectroscopic analysis was performed using a Vector 22 Fourier transform infrared spectrometer (FT-IR), and the pH of the sample was tested using an Orion pH meter. All reagents used were analytical reagents. Vessels were immersed in diluted HCl for 12 h before use, washed using ordinary water, and then washed a further four times using DI water and then dried for future use. Parallel experiments (three) were designed, and the mean values were obtained for data analysis.

### Models and equations

The pseudo-first-order kinetic equation and pseudo-second-order kinetic equation were used to fit the adsorption results, and the equations are as follows [18,20]:

Pseudo-first-order kinetic equation:

$$Log\;\left(Q_{e}–Q_{t}\right)=logQ_{e}–k_{1}t/2.303$$ (2)

Pseudo-second-order kinetic equation:

$$t/Q_{t}=1/\left(k_{2}Q_{e}{}^{2}\right)+t/Q_{e}$$ (3)

where Q_t_ and Q_e_ are the adsorption amounts of Pb^2+^ (mg·g^−1^), at the moment t and when adsorption equilibrium was reached, respectively. In addition, t represents the adsorption time (h^−1^), while k_1_ and k_2_ (mg·g^−1^·h^−1^) are reaction rate constants in the pseudo-first-order dynamic model and pseudo-second-order dynamic model, respectively.

Langmuir and Freundlich adsorption isotherm equations are often used to describe the adsorption of heavy metal ions by adsorbents, soils and mineral components, and the equations are [21,22]:

Langmuir equation: *Q*_*e*_ *= bQ*_*m*_*C*_*e*_/(1 *+ bC*_*e*_)

Freundlich equation: *Q*_*e*_ *= K*_*f*_*C*_*e*_^*N*^

where *C*_*e*_ is the Pb^2+^ concentration (mg·L^−1^) of the solution in an adsorption state; *Q*_*m*_ is the maximum adsorption amount (mg·g^−1^); b is a parameter (L·mg^−1^) characterizing the affinity between the adsorbent and adsorbate. Specifically, a larger b value corresponds to a larger adsorption affinity. The parameter *K*_*f*_ is the Freundlich adsorption isotherm (mg^1−N^·g^−1^·L^−N^) and N is the Freundlich index.

### Statistical analysis

The experimental data were calculated by Excel 2013 software (shown as means ± S.E.) followed by statistical calculation using SPSS 20.0 software with one-way analysis of variance at a 0.05 probability level.

## Results and discussion

### Adsorption kinetics of alum plasma in removing Pb^2+^

**Fig 1:**
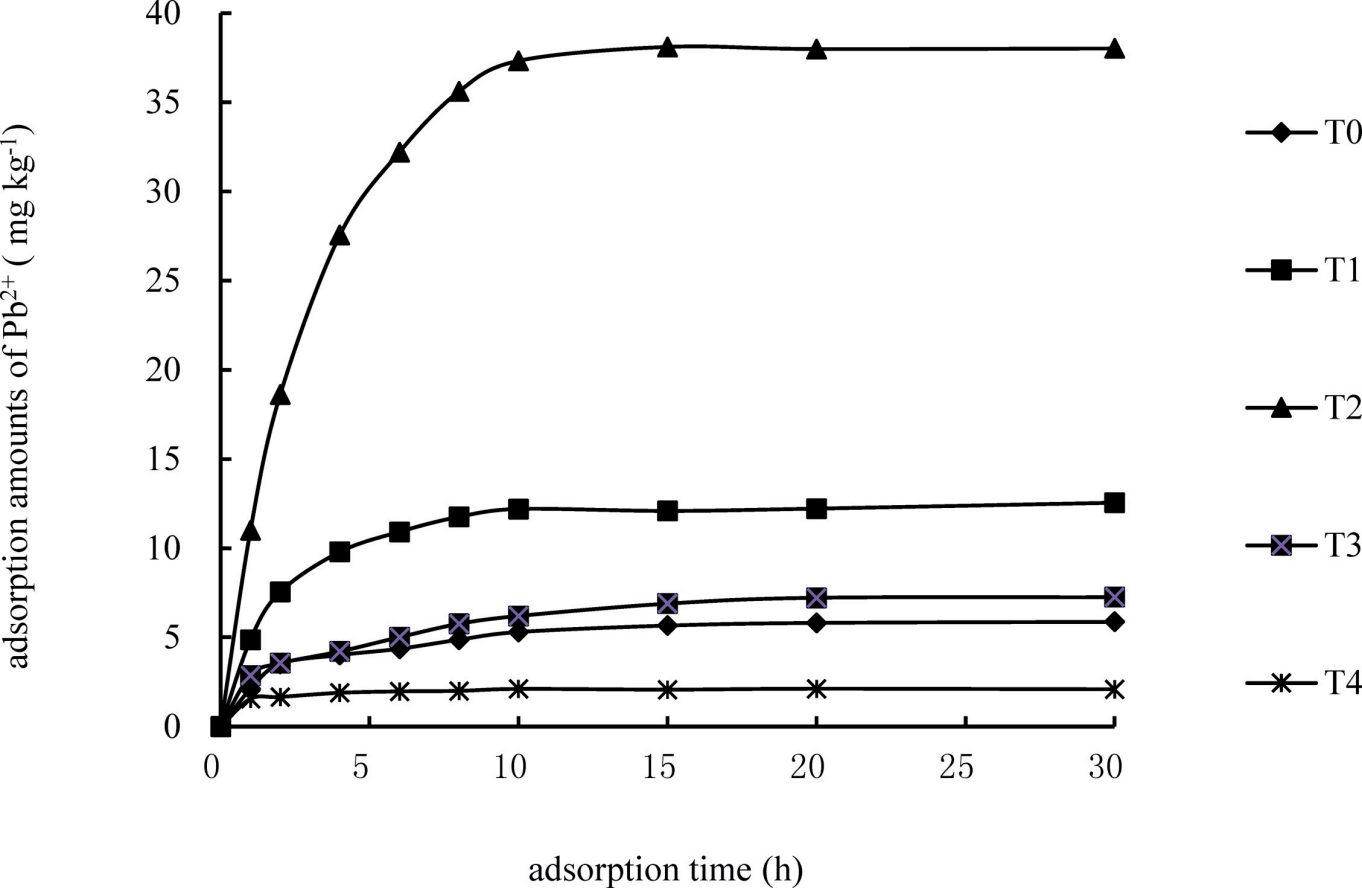
Adsorption dynamic curves of Pb^2+^ by ameliorated alum plasma and original alum plasma (Initial Pb^2+^ concentration in the solution is 20 mg·L^−1^; and the initial pH is 6.0).

The dependence of the Pb^2+^ amount adsorbed by ameliorated alum plasma with time is shown in Fig 1. During the initial 5 h, the adsorption rates of the five types of alum plasma were relatively high and then rapidly slowed down to a stabilized value. After 10 h, the adsorption amount of Pb^2+^ cannot increase significantly, indicating that the adsorption of Pb^2+^ by ameliorated alum plasma had reached a balanced state.

The model-fitting results are listed in Table 1. As observed in Fig 1 and Table 1, it is evident that the results obtained from the pseudo-first-order dynamic model are superior, exhibiting the highest coefficient of determination R^2^ (Table 1), which best corroborates the measured data points. The adsorption rate constant fitted using the pseudo-first-order dynamic model reflects the reaction rate of the adsorption process. The larger the dynamic rate constant is, the shorter the time it takes for the adsorption process to reach an equilibrium state [23]. As indicated in Fig 1, the k_1_ values of the types of alum plasma are significantly different, suggesting that the adsorption rate constants of Pb^2+^ by alum plasma differ with the different amelioration methods used. Compared to the T0 (0.155 h^−1^), the adsorption process of Pb^2+^ in T2 hastened (0.228 h^−1^), and the adsorption processes of Pb^2+^ in T3 and T4 decelerated, with values of 0.147 h^−1^ and 0.109 h^−1^, respectively. The equilibrium adsorption capacities of the T0, T1, T2, T3 and T4 were 5.07 ± 0.8, 12.55 ± 1.5, 40.6 ± 3.1, 7.25 ± 0.7 and 2.09 ± 0.7 mg·g^−1^, respectively (Fig 1).

**Table 1:** The fitting parameters of the pseudo-first-order kinetic equation and pseudo-second-order kinetic equation for Pb^2+^ adsorption by ameliorated alum plasma and original alum plasma.

Sample	Pseudo-first-order kinetic equation			Pseudo-second-order kinetic equation		
	Q_e_/(mg·g^−1^)	k_1_/h^−1^	R^2^	Q_e_/(mg·g^−1^)	K_2_/(mg·g^−1^·h^−1^)	R^2^
T0	5.07	0.155	0.841	8.6	0.0137	0.846
T1	12.55	0.171	0.966	18.4	0.00443	0.952
T2	40.6	0.228	0.984	55.9	0.00375	0.968
T3	9.6	0.147	0.812	10.7	0.00241	0.799
T4	2.8	0.109	0.804	3.1	0.00742	0.811

### Isothermal adsorption of Pb^2+^ by adsorbents

**Fig 2:**
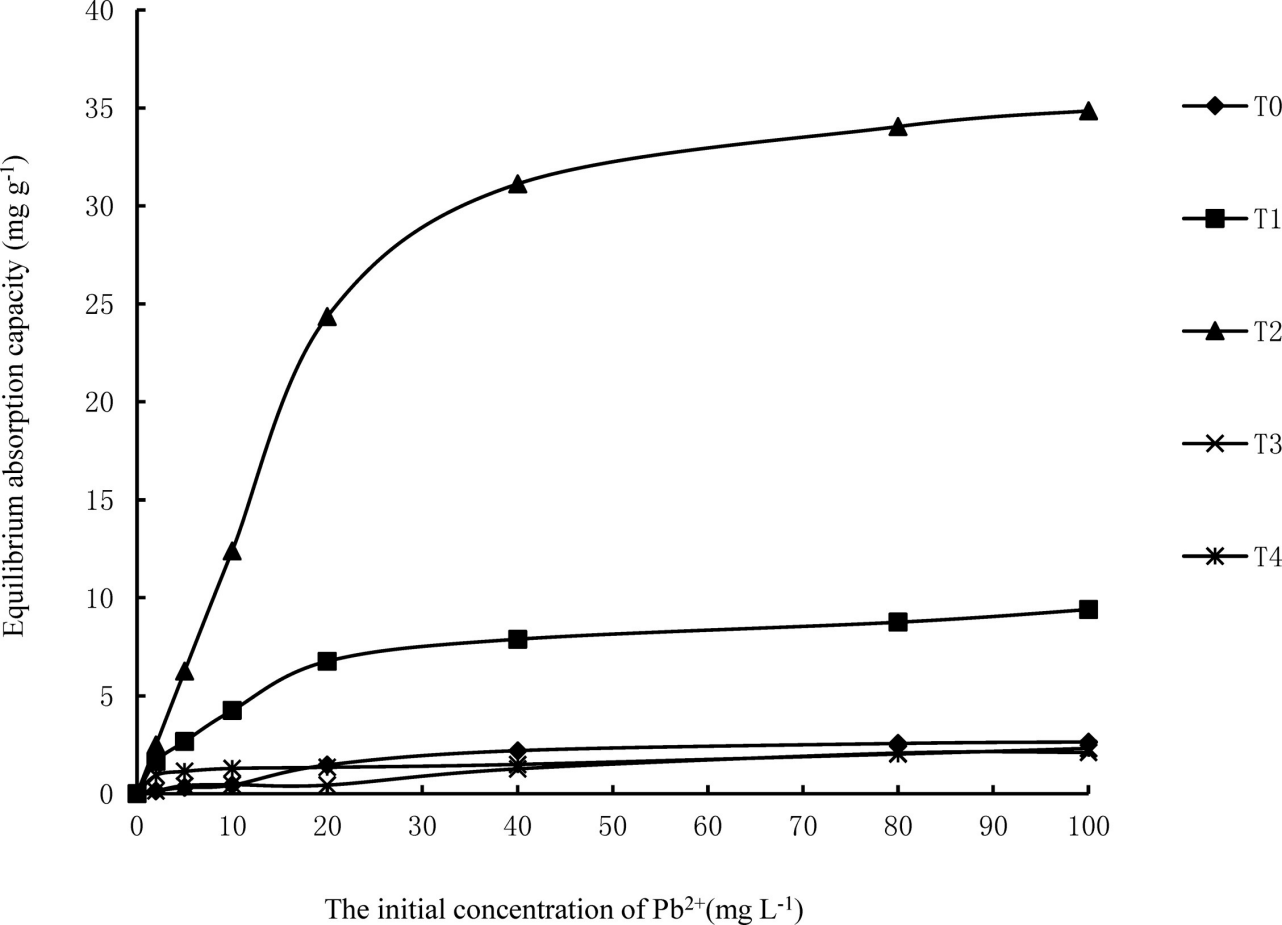
The adsorption isotherms and adsorption rate curves of Pb^2+^ by the original alum plasma and ameliorated alum plasma (pH = 6, 25°C).

Fig 2 presents the isothermal adsorption curves of Pb^2+^ of the five types of ameliorated alum plasma. Evidently, the adsorption of Pb^2+^ by ameliorated alum plasma is related to the concentration of Pb^2+^ in its equilibrium liquid. At low concentrations (0–10 mg·L^−1^), the adsorption of Pb^2+^ rapidly increased with an increase in the solution concentration, and in particular, the T2 revealed a dramatic increase in the adsorption of Pb^2+^. When the solution concentration was equal to or higher than 40 mg·L^−1^, the adsorption of Pb^2+^ tended to approach an equilibrium value. The T2 reported the highest adsorption ability of Pb^2+^, followed by T1, while T3 and T4 have similar Pb^2+^ adsorption abilities as T0.

The fitting parameters using the Langmuir and Freundlich equations are listed in Table 2. Results indicate that both the Langmuir and Freundlich equations can sufficiently describe the adsorption isotherms of alum plasma. While Langmuir fits the adsorption data of the different types of alum plasma well, the Freundlich equation only fits the adsorption data of the hot alkali ameliorated alum plasma. As a result, it is better to apply Langmuir when discussing the adsorption capability of T2.

**Table 2:** The fitting results of Pb^2+^ adsorption by ameliorated alum plasma using Langmuir and Freundlich equations.

Sample	Langmuir equation			Freundlich equation		
	Q_m_/(mg·g^−1^)	b/(L·mg^−1^)	R^2^	K_f_/(mg^1−N^·g^−1^·L^−N^)	N	R^2^
T0	8.8	0.201	0.916	9.8	0.442	0.751
T1	18.9	1.047	0.978	20.9	0.231	0.82
T2	57.3	1.297	0.992	63.3	0.218	0.902
T3	10.9	0.126	0.902	12.1	0.156	0.748
T4	3.2	0.078	0.814	3.5	0.247	0.711

As shown in Fig 2 and Table 2, the T2 indicated the largest Pb^2+^ adsorption amount (Q_m_) and the highest adsorption affinity (b), followed by T1. The maximum adsorption quantities of Pb^2+^ in T2 and T1 were 57.3 and 18.9 mg·g^−1^, respectively, which was 6.51 and 2.14 times the maximum adsorption amount of the T0 (8.8 mg·g^−1^). The adsorption affinity parameters b for Pb^2+^ in T2 (1.247 L·mg^−1^) and T1 (1.091 L·mg^−1^) were larger than in T0 (0.201 L·mg^−1^). The adsorption affinity parameter b for Pb^2+^ inT4 was significantly smaller (0.0781 L·mg^−1^) than the T0, while the adsorption affinity parameter b for Pb^2+^ in the T3 indicated no apparent difference compared to T0.

### The influence of pH on Pb^2+^ adsorption by ameliorated alum plasma

**Fig 3:**
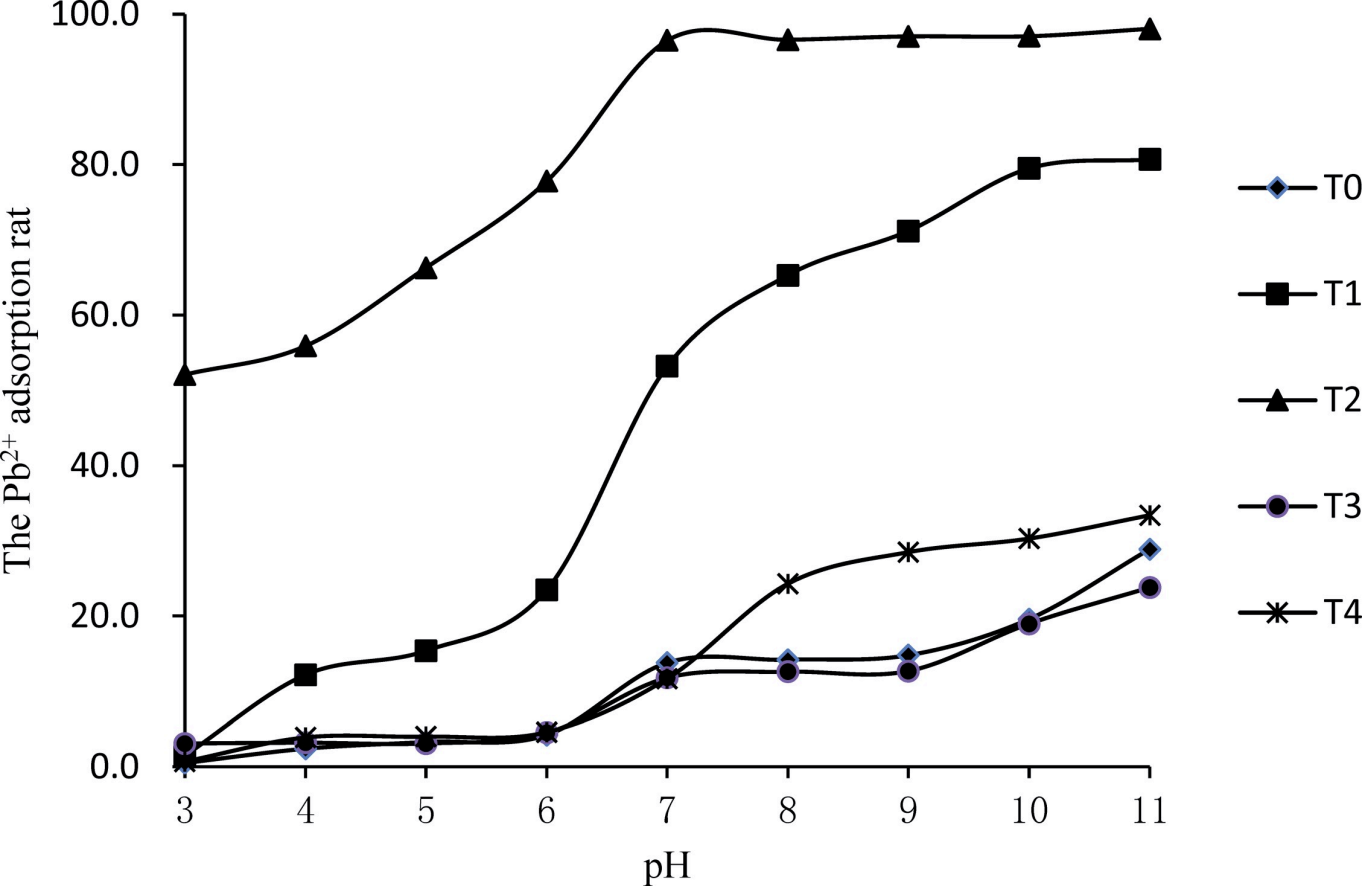
The influence of pH in the solution on the removal of Pb^2+^ by ameliorated alum plasma.

The relationship between the Pb^2+^ adsorption rates of the five different types of alum plasma along with the equilibrium pH in a solution is shown in Fig 3. It is evident that pH is a primary factor influencing Pb^2+^ adsorption by ameliorated alum plasma. When the pH of the solution was within 3.0–7.0, the Pb^2+^ adsorption rate of T1 and T2 significantly increased along with an increase in pH. When the pH of the solution was higher than seven, the Pb^2+^ adsorption rate of T2 was close to saturation, and was only slightly influenced by an increase in pH. When the pH was between 7.0–9.0 in the T3 and the T0, the Pb^2+^ adsorption rates tended to be stable. However, when the pH was higher than nine, the Pb^2+^ adsorption rate increased with the increase of the pH value. The Pb^2+^ adsorption rate by T1 significantly increases with the increase of the pH value.

Therefore, when the pH is lower than six, it becomes difficult for alum plasma to adsorb Pb^2+^, which is consistent with previous research [24]. This is probably due to the fact that the particle surfaces adsorb more H^+^, in place of Pb^2+^, and the negative surface charge decreases accordingly there by substantially lowering the cohesion between ameliorated alum plasma and Pb^2+^. With an increase in pH, the H^+^ concentration in the solution decreases, thus decreasing the influence of H^+^ on competitive Pb^2+^ adsorption, creating more opportunities for Pb^2+^ to interact with the surface active sites.

### The influence of ameliorated alum plasma on the effective Pb content in soils

All five types of alum plasma studied in this paper were able to decrease the effective content of Pb in soils and lower the migration ability of Pb in the environment. The different amelioration methods also indicated significant differences regarding the effective contents of Pb in soils. In Table 3, it is evident that the influence of the five alum plasma adsorbents on the effective content of heavy metal Pb in soils obeys the following order: T2 > T1 > T0 > T4 > T3. For the same kind of ameliorated alum plasma, along with an increase in fortification concentration and an elongation of the storage period, the effective content of heavy metal Pb in soils reduces. For the same adsorption, the fortification concentration and passivation time both have a significant influence on the effective content of Pb in soils. When the passivation time is fixed, the T2 with 5.0% fortification indicated the lowest effective Pb content. For example, when the passivation time was 4 weeks, the T2 with 5.0% fortification indicated a lowest effective Pb content (25.17 mg·kg^−1^), 69.0% lower than the control sample (81.78 mg·kg^−1^). When the passivation time was 8 weeks, the T2 with 5.0% fortification revealed a lowest effective Pb content of 21.7 mg·kg^−1^, 65.3% lower than the control sample (62.6 mg·kg^−1^). When the passivation time was 16 weeks, the T2 with 5.0% fortification indicated a lowest effective Pb content of 19.87 mg·kg^−1^, 60.8% lower than the control sample (50.8 mg·kg^−1^).

**Table 3:** The influence of four ameliorated alum plasmas on the effective content of Pb in soils (mg·kg^−1^, mean ± standard deviation, n = 3).

Adsorption type	Amount added%	Effective Pb content mg·kg^−1^		
		4 week	8 week	16 week
CK	0.0	81.78 ± 2.0	62.6 ± 0.5	50.8 ± 1.3
T0	0.1%	60.25 ± 1.3^a^	55.25 ±1.3^a^	37.92 ± 1.8^a^
	0.2%	51.64 ± 1.2^b^	44.41 ± 1.2^b^	37.88 ± 1.3^a^
	0.5%	45.19 ± 2.3^c^	44.11 ± 2.3^b^	35.90 ± 1.9^a^
	1.0%	43.26 ± 2.1^c^	41.53 ± 0.9^b^	34.34 ±1.9^a^
	2.0%	41.85 ± 1.9^c^	40.73 ± 1.5^b^	33.86 ± 1.5^a^
	5.0%	36.51 ± 1.5^d^	28.71 ± 1.7^c^	28.87 ± 0.9^b^
T1	0.1%	58.80 ± 2.1^a^	50.79 ± 1.6^a^	45.27 ± 2.0^a^
	0.2%	41.97 ± 2.5^b^	47.59 ± 2.8^b^	35.10 ± 0.7^b^
	0.5%	41.66 ± 0.9^b^	47.12 ± 2.4^b^	35.02 ± 0.9^b^
	1.0%	41.17 ± 0.8^b^	46.80 ± 1.8^b^	34.17 ± 0.5^b^
	2.0%	41.16 ± 1.2^b^	46.75 ± 0.8^b^	33.81 ± 1.1^b^
	5.0%	39.75 ± 0.9^c^	38.85 ± 1.4^c^	31.96 ± 1.8^c^
T2	0.1%	66.46 ± 2.0^a^	47.79 ± 1.6^a^	33.58 ± 0.9^a^
	0.2%	50.26 ± 3.1^b^	43.56 ± 1.8^ab^	31.77 ± 0.5^a^
	0.5%	37.93 ± 2.9^c^	40.24 ± 1.1^b^	31.53 ± 1.3^a^
	1.0%	34.29 ± 1.8^c^	31.43 ± 1.2^c^	28.39 ± 1.6^ab^
	2.0%	30.12 ± 1.5^c^	26.50 ± 1.1^d^	25.05 ± 0.5^b^
	5.0%	25.17 ± 1.2^d^	21.70 ± 1.4^e^	19.87 ± 0.7^c^
T3	0.1%	62.30 ± 1.4^a^	61.60 ± 0.9^a^	45.86 ± 0.8^a^
	0.2%	53.96 ± 0.8^b^	55.31 ± 0.8^b^	44.45 ± 1.5^ab^
	0.5%	50.77 ± 0.5^b^	53.34 ± 0.5^b^	40.13 ± 1.7^b^
	1.0%	48.36 ± 1.3^b^	51.76 ± 1.2^b^	39.43 ± 0.8^b^
	2.0%	45.48 ± 2.1^c^	46.19 ± 2.3^c^	36.68 ± 0.6^c^
	5.0%	42.78 ± 2.4^c^	45.11 ± 1.5^c^	36.30 ± 3.2^c^
T4	0.1%	71.40 ± 1.7 ^a^	58.61 ± 2.5 ^a^	47.96 ± 2.6 ^a^
	0.2%	59.66 ± 2.4 ^b^	53.57 ± 0.73 ^a^	43.30 ± 0.8 ^a^
	0.5%	55.11 ± 3.0 ^c^	52.64 ± 0.95 ^a^	36.03 ± 1.2 ^b^
	1.0%	54.28 ± 0.8 ^c^	47.53 ± 0.87^ab^	36.22 ± 2.3 ^b^
	2.0%	44.07 ± 1.6 ^d^	41.60 ± 1.1 ^c^	33.03 ± 1.8 ^c^
	5.0%	41.79 ± 1.2 ^d^	41.56 ± 1.5 ^c^	31.52 ± 1.5 ^c^

Note: the letters following digits in the table represent the variance of effective Pb content for different adsorbents with varying addition amounts at the same culture time, p < 0.05.

A adsorption can convert heavy metals that are harmful to the environment to a stable state that cannot easily be utilized by plants or animals, by methods of adsorption and co-precipitation [25]. Alum plasma contains large amounts of aluminum silicate clay minerals, iron-aluminum oxides, and the corresponding hydrates. Many investigations have shown that clay minerals and iron-aluminum oxides can adsorb and immobilize the heavy metal Pb^2+^, and the addition of alum plasma to soils not only improves the adsorption and immobilization ability of Pb^2+^ by the soils, but also lowers the Pb^2+^ concentration in soil solutions, thus influencing the bio-availability of the heavy metal Pb [26]. For example, Usman et al. [27] applied iron oxide and clay as adsorbents in order to study their influence on the bioavailability of Zn, Cd, Cu, Ni and Pb in sludges [28]. The results indicated that the introduction of adsorbents significantly inhibited the bioavailability of Zn, Cd, Cu, Ni and Pb in sludges. Iron, manganese, aluminum and silicon oxides (i.e. silicate minerals) with layered, frame-shaped, or porous structures present channel characteristics and chemical adsorption, especially oxides and hydroxides of valence-variable elements including Fe, Mn, and Al, which contain certain oxidation-reduction properties. Upon adsorbing heavy metals, precipitates or co-precipitates of calcium silicoaluminate may form, resulting in adhesion and forming further aggregates with other mineral particles in soils to immobilize the heavy metal ions in the crystal layer, with the result that the heavy metal ions then become a stable state that isdifficult to release and is not utilizable by plants [26,29,30].

There are various uncertainties that influence the heavy metal adsorbing/immobilizing abilities of adsorbents in soils, for example, soil type, soil property, Pb^2+^ content in soil solution and environmental factors [9]. This study revealed that all five types of alum plasma are capable of adsorbing and stabilizing Pb^2+^ in soils, thus lowering the effective Pb^2+^ content. With an increase in concentration and elongation of the storage period, the effective heavy metal Pb content in soils decreases for all types of ameliorated alum plasma. In particular, the T2 is the most effective in reducing the content of effective Pb in soils, and the maximum reduction rate can reach 69.0%. Thus, the introduction of T2 may lower the content of effective Pb^2+,^ restoring polluted soils. The passivation storage mechanism may require further investigation.

### Surface property characterization

The equilibrium adsorption capacity (Q_e_) of the adsorbent per unit mass is influenced by various factors, including the physical structure (surface structure) of the adsorbent, chemical composition, concentration of the adsorbate in the fluid phase, operation temperature, etc. [18]. This investigation revealed that the amount of Pb^2+^ absorbed by the five different types of ameliorated alum plasma all increased with an increase in Pb^2+^ mass concentration in the equilibrium liquid. In Fig 2, it is apparent that, in comparison with the other four ameliorated alum plasmas, the T2 indicated the highest equilibrium adsorbent value of Pb^2+^, and the adsorption isotherm best fit to the Langmuir equation (*R*^2^ = 0.992). This suggests that the T2 adsorbs Pb^2+^ mainly via monolayer adsorption. The maximum Pb^2+^ adsorption (Table 2) by T2 was 57.3 mg·g^−1^, which was 6.51 times than the T0, 3.03 times the T1 and 17.9 times the T4, indicating that following hot alkali amelioration treatment, the performance of alum plasma absorbing Pb^2+^ is significantly improved.

**Fig 4:**
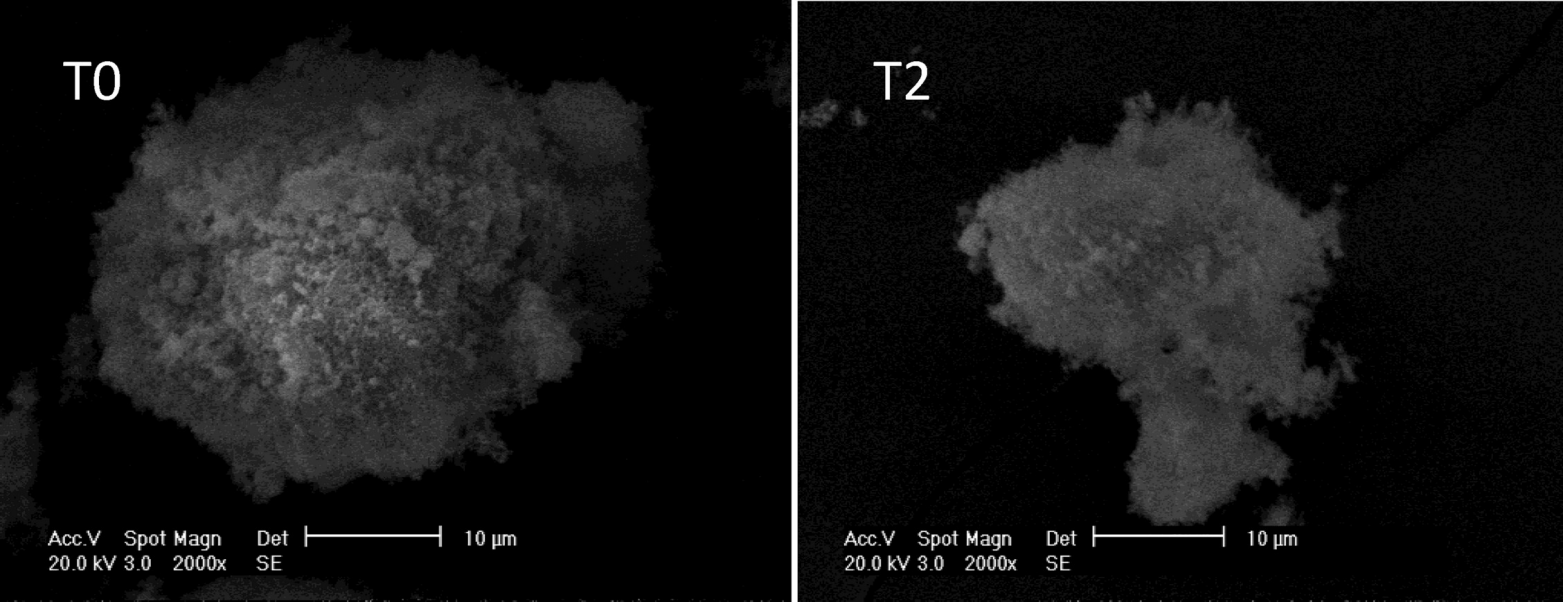
SEM images of the original alum plasma and hot alkali ameliorated alum plasma (200 kV, 2000x).

The SEM images of T0 and T2 (Fig 4) indicate that the external surface of the T2 is rough and porous, and its surface structure is loose, presenting smaller particles compared to the T0. This suggests that during the hot alkali amelioration, a surface erosion phenomenon occurs. Correspondingly, the BET results indicated that the specific area of T2 increased from 15.5 m^2^·g^−1^ to 35.3 m^2^·g^−1^, which was 127.7% higher than that before amelioration. According to Ogura et al. [31], following alkali amelioration, the micro-pores of zeolite significantly increase, which is comparable to the results of this paper [31]. Following hot alkali treatment, the alum plasma releases microcellular structures of silica-alumina mixed oxides once occupied by other ions, thus increasing the specific area. From adsorption dynamics, it is concluded that Pb^2+^ adsorption by hot alkali alum plasma primarily occurs inside the micro pores, and the equilibrium adsorption amount is influenced by the spreading of the internal pore channel. The increase of the aperture enhances the diffusion of pollutants into the pore channels and the resulting adsorption effect. Consequently, the modification of surface properties of T2 benefits Pb^2+^ adsorption and precipitation.

**Fig 5:**
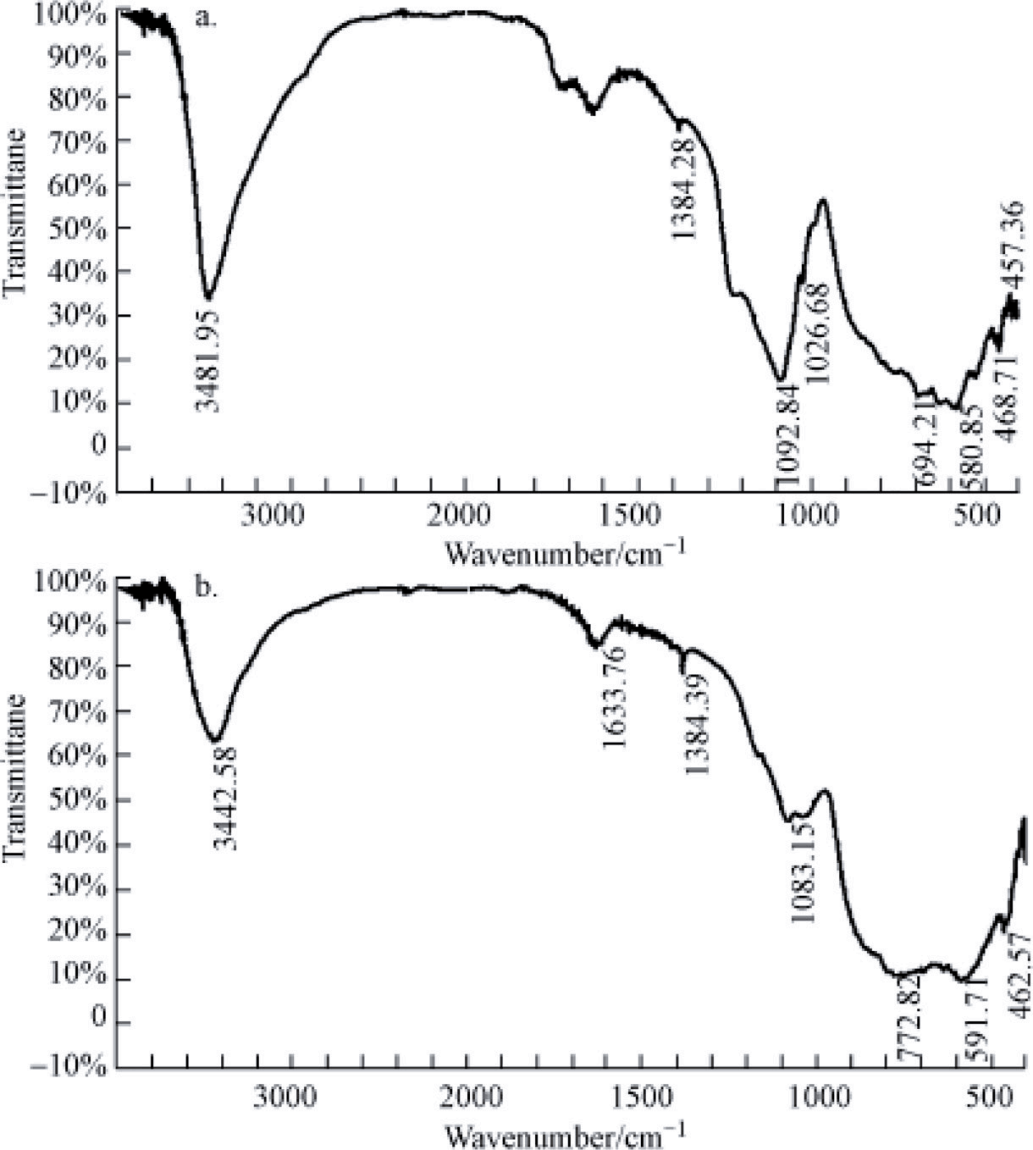
The IR adsorption spectra of original alum plasma (a) and hot alkali ameliorated alum plasma (b).

The IR spectra of T0 and T2 are shown in Fig 5. The location and shape of the characteristic peaks showed little change, indicated by the appearance of a relatively wide adsorption peak at 3481 and 3442 cm^−1^, the appearance of which was due to the stretching vibration of O-H, indicating that both of the two types of alum plasmas contained typical crystal structures. They differ in that following hot alkali amelioration, the adsorption peak at 1092 cm^−1^ (belonging to Si-O-Si stretching vibration adsorption peak) disappeared, while the corresponding peak at 1083 cm^−1^ became sharper, indicating that portions in the T2 are selectively dissolved, and the alkali amelioration lowers the crystallinity making the internal structure irregular. Moreover, the symmetry lowers, and the vibration frequencies of functional groups are no longer limited to certain values. This is indicated by the fact that the adsorption width widens and the number of spectral bands decreases. Since the original alum plasma contains crystalline silicates or silica-aluminate, the silica tetrahedron or alumina tetrahedron form pores and cavity systems via oxo-bridged oxygen bonding. Following hot alkali treatment, the Si^4+^ in silica tetrahedron is replaced with Al^3+^, resulting in superfluous negative charges, replaced by alkaline earth metal cations. These replaced cations have a high degree of freedom and ion-exchangeable characteristics, which enables them to undergo adsorption or an ion-exchange process with cations in the solution. As a consequence, the adsorption and removal of Pb^2+^ increases following hot alkali treatment of alum plasma.

## Conclusion

Of the four amelioration methods used for the treatment of alum plasma, the T2 significantly improved the adsorption/passivation of Pb^2+^ by alum plasma. The experimental results of adsorption dynamics indicate that the Pb^2+^ adsorption by T2 satisfies the pseudo-first-order kinetic and Langmuir equations. The maximum adsorption amount was found to be 6.51 times higher than the original adsorption amount. The soil culture experimental results indicated that the T2 significantly lowered the effective Pb content in soils, with a reducing amount of 69.0% compared to the control sample. As a consequence, the T2 requires further investigation as a novel adsorption to restore soils polluted by Pb.

## Supporting information

S1 Fig SEM images of hot acidic ameliorated alum plasma (T1, 200 kV, 2000x). (TIF)

S2 Fig SEM images of high-temperature calcinated alum plasma (T3, 200 kV, 2000x). (TIF)

S3 Fig SEM images of sulf-hydrylated alum plasma (T4, 200 kV, 2000x). (TIF)

S1 Table The raw data in Figs 1–3. (XLS)
