# Supplementary figures and images for: Adsorption of Pb2+ by ameliorated alum plasma in water and soil
Source: PLoS One. 2019 Jan 25;14(1):e0210614. doi: 10.1371/journal.pone.0210614 (PMC6347192; doi:10.1371/journal.pone.0210614)

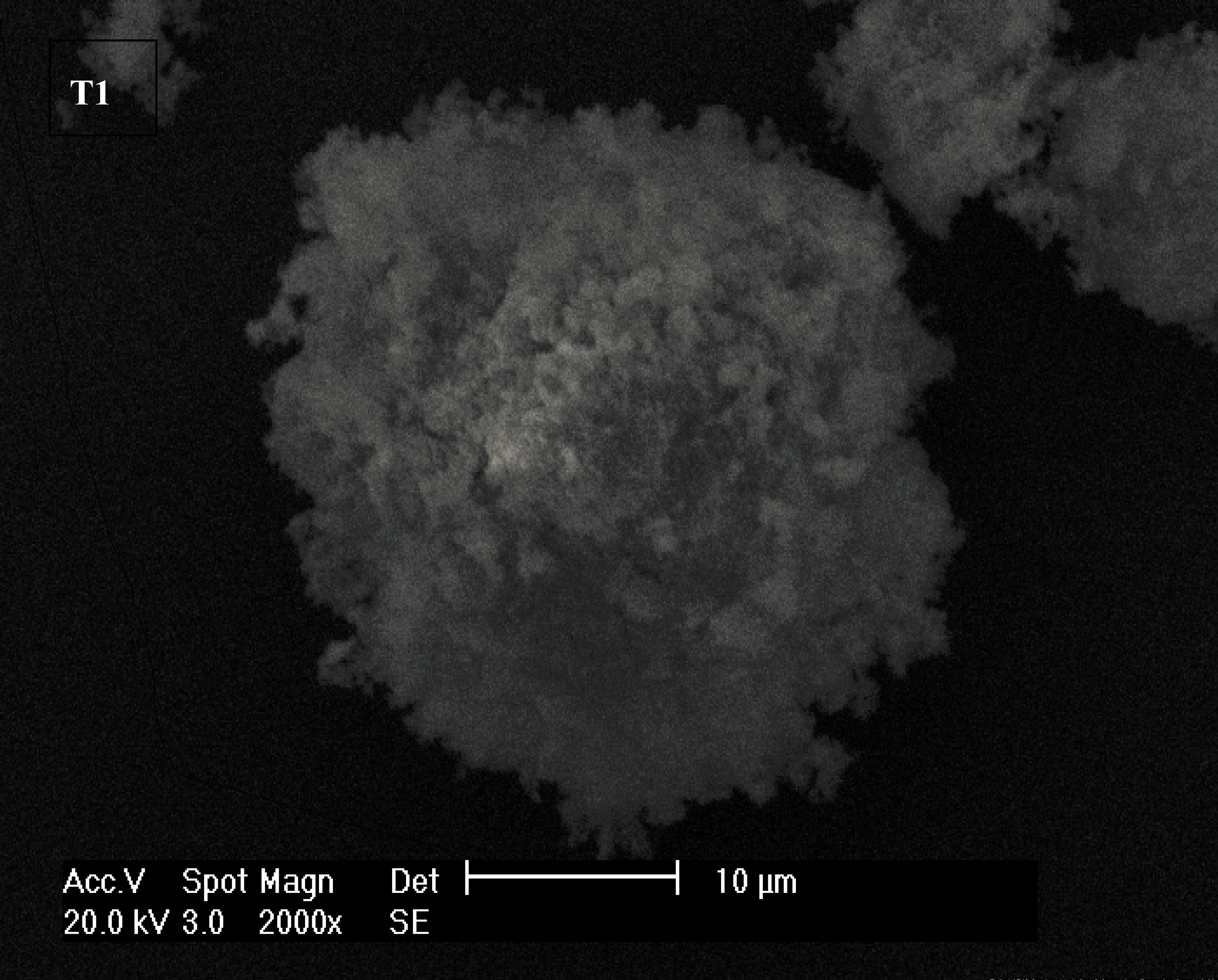

Supplement: S1 Fig — (TIF) [file pone.0210614.s001.tif]

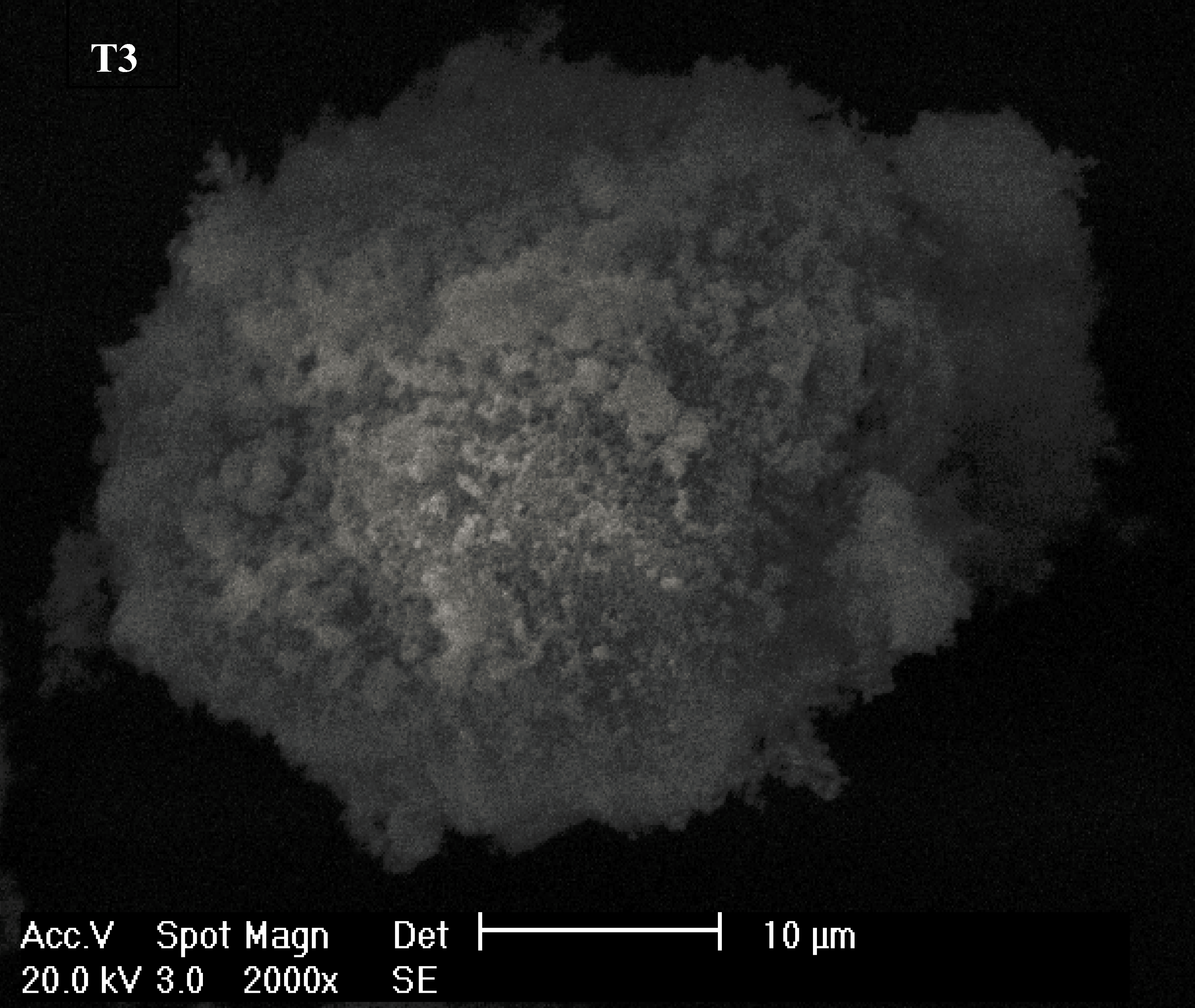

Supplement: S2 Fig — (TIF) [file pone.0210614.s002.tif]

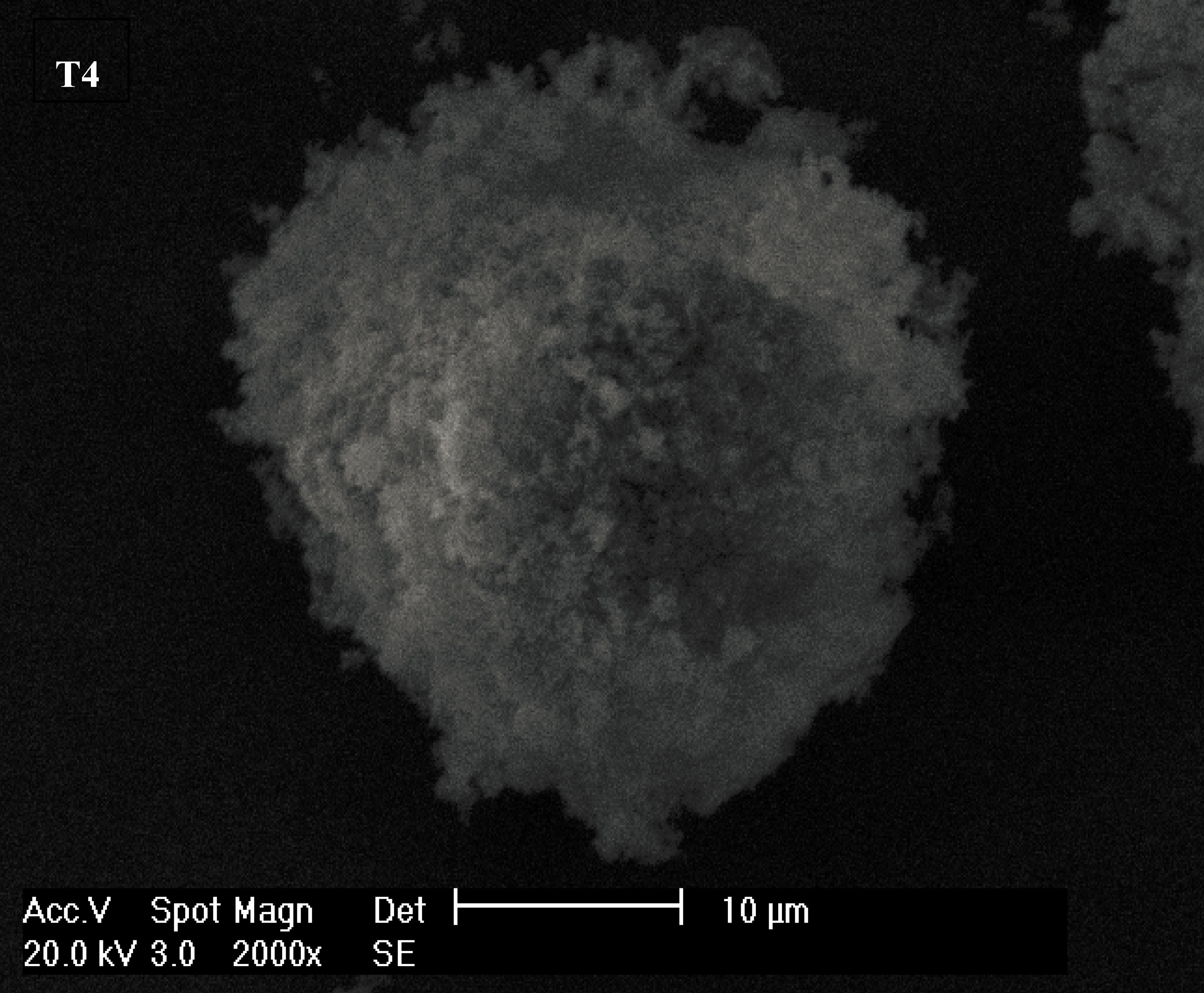

Supplement: S3 Fig — (TIF) [file pone.0210614.s003.tif]
